# Is There an Association Between Cigarette Smoking and Advanced Liver Fibrosis in Smokers with HIV, Heavy Drinking and High Prevalence of HCV?

**DOI:** 10.3390/jcm14041169

**Published:** 2025-02-11

**Authors:** Daniel Fuster, Kaku So-Armah, Debbie M. Cheng, Elena Blokhina, Gregory Patts, Dmitry Lioznov, Natalia Gnatienko, Michelle T. Long, Matthew S. Freiberg, Hilary Tindle, Jeffrey H. Samet

**Affiliations:** 1 Addiction Unit, Internal Medicine Department, Hospital Universitari Germans Trias i Pujol, Universitat Autònoma de Barcelona, 08916 Badalona, Spain; 2 Clinical Addiction Research and Education Unit, Section of General Internal Medicine, Department of Medicine, Boston University School of Medicine/Boston Medical Center, Boston, MA 02118, USA; kaku@bu.edu (K.S.-A.); jsamet@bu.edu (J.H.S.); 3 Biostatistics Department, Boston University School of Public Health, Boston, MA 02118, USA; dmcheng@bu.edu; 4 Laboratory of Clinical Psychopharmacology of Addictions, First Pavlov State Medical University, 197022 St. Petersburg, Russia; blokhinaelena@gmail.com; 5 Biostatistics and Epidemiology Data Analytics Center, Boston University School of Public Health, Boston, MA 02118, USA; gpatts@bu.edu; 6 Department of Infectious Diseases and Epidemiology, First Pavlov State Medical University, 197022 St. Petersburg, Russia; dlioznov@yandex.ru; 7 Smorodintsev Research Institute of Influenza, 197022 St. Petersburg, Russia; 8 Clinical Addiction Research and Education Unit, Section of General Internal Medicine, Department of Medicine, Boston Medical Center, Boston, MA 02118, USA; natalia.gnatienko@bmc.org; 9 Gastroenterology Section, Department of Medicine, Boston University School of Medicine/Boston Medical Center, Boston, MA 02118, USA; mtlong@bu.edu; 10 Cardiovascular Medicine Division, Vanderbilt University Medical Center, Nashville, TN 37232, USA; matthew.s.freiberg@vumc.org; 11 ViTAL, the Vanderbilt Center for Tobacco, Addiction and Lifestyle, Vanderbilt University Medical Center, Nashville, TN 37232, USA; hilary.tindle@vumc.edu; 12 Department of Community Health Sciences, Boston University School of Public Health, Boston, MA 02118, USA

**Keywords:** tobacco use, liver fibrosis, alcohol, hepatitis C virus, HIV

## Abstract

**Background:** Cigarette smoking has been associated with liver fibrosis in the setting of hepatitis C virus (HCV) infection but has not been studied among people with HIV (PWH) who consume alcohol. **Methods:** This is a cross-sectional study of PWH with heavy drinking and daily smoking in St. Petersburg, Russia. The primary independent variable was past 30-day cigarettes per day (cpd), and the secondary independent variable was pack-years at study entry. Advanced liver fibrosis was defined as FIB-4 > 3.25. Analyses were adjusted for gender, body mass index (BMI), past 30-day number of heavy drinking days, HCV and CD4 count. **Results:** Participants (n = 400) were two-thirds male (67.3%), young (median age 38 years), lean (median BMI 22), HCV antibody positive (84.5%) and not severely immune suppressed (median CD4 count 351). The median number of past-month cpd was 20 (IQR: 15–25), and the median pack-years was 24 (IQR: 17–31.8). The prevalence of advanced liver fibrosis was 11.3% (45/400). In the adjusted logistic regression analyses, we did not observe a significant association between cpd [middle (10.1–20 cigarettes) vs. lowest (5–10 cigarettes) category (adjusted odds ratio [aOR] (95% confidence interval [CI]): 1.06 (0.40–2.83), highest (>20.0 cigarettes) vs. lowest category aOR (95% CI): 0.65 (0.21–1.99), global *p*-value = 0.62]. The secondary analysis with pack-years yielded similar results [middle (20.1–30 pack-years) vs. lowest category (≤20 pack-years) aOR (95% CI): 0.81 (0.33–1.99), highest category (>30 pack-years) vs. lowest category aOR (95% CI): 0.91 (0.38–2.19); global *p*-value = 0.58]. **Conclusions:** In this Russian cohort of PWH, we did not detect an association between recent cigarette use or mean pack-years and advanced liver fibrosis.

## 1. Introduction

Liver disease is a major concern among persons living with HIV (PWH), and liver-related deaths are a leading cause of non-AIDS mortality, especially among patients who present other risk factors for liver disease [1,2]. Coinfection with hepatitis C virus (HCV) is common in PWH with prior or active injection drug use [2,3]. Chronic HCV infection in the absence of antiviral therapy is associated with progressive liver fibrosis [4]. Liver fibrosis is the main predictor of the evolution to end-stage liver disease in both chronic HCV infection and other forms of chronic liver disease, such as alcohol-associated liver disease [5,6,7]. Several cofactors are associated with increased progression of liver fibrosis in HCV-infected patients, such as age, duration of HCV infection, obesity, unhealthy alcohol use, coinfection with HIV or hepatitis B, and immune suppression [2,8].

Cigarette smoking has been associated with liver fibrosis in the setting of hepatitis C by some researchers [9,10], while others have failed to find an association [11,12]. Cigarette use appears to have a negative impact on hepatitis B-related and alcohol-related cirrhosis of the liver and is regarded as a predisposing cofactor for primary biliary cirrhosis [10]. In addition, cigarette smoking is a carcinogen that has been associated with an increased incidence of hepatocellular carcinoma [13]. Findings in cohorts of PWH have also been mixed [12,14], and the possible association between tobacco and liver disease has not been studied among PWH with co-occurring unhealthy alcohol consumption. A recently published review summarizes the impact of cigarette smoking and different forms of liver disease [15]. Among the potential mechanisms for fibrogenesis are inflammatory activity, bacterial translocation, oxidative stress, stellate cell activation and endothelial dysfunction [15].

Despite the fact that direct-acting antiviral (DAA) agents have changed the landscape of chronic HCV infection, and while active alcohol use is no longer a formal limitation for its prescription [16], the use of DAA agents is still low in Russia [17]. Russian PWH have many other cofactors that are associated with progressive liver disease, like unhealthy alcohol use [7] and suboptimal prescription of antiretroviral therapy [18]. In addition, the prevalence of cigarette smoking is higher among PWH in comparison to the general population [19]. In particular, tobacco use is very high among Russian PWH who drink alcohol, which is why the study of tobacco use as a potentially modifiable risk factor for the occurrence of liver fibrosis is an opportunity in this setting.

In the present study, we aimed to examine the association between levels of cigarette use and advanced liver fibrosis in a cohort of smokers at high risk for liver disease: Russian PWH with heavy alcohol use and a high prevalence of HCV coinfection.

## 2. Materials and Methods

### 2.1. Participants

This study is an exploratory cross-sectional analysis of baseline data in the St PETER HIV study, a controlled trial conducted to compare the effects of two partial nicotinic receptor agonists, varenicline and cytisine, on alcohol consumption, alcohol craving, smoking, inflammation, coronary heart disease risk and mortality risk among PWH with heavy drinking and daily smoking in St. Petersburg, Russia [20].

Participants were recruited between July 2017 and December 2019 from a cohort study [21], as well as from HIV clinical care sites and non-clinical sites, and via snowball recruitment in St. Petersburg, Russia.

### 2.2. Eligibility

Eligibility criteria for the St PETER HIV study included the following: 18–70 years old; HIV-positive; ≥5 heavy drinking days (i.e., National Institute on Alcohol Abuse and Alcoholism [NIAAA] at-risk drinking levels, that is, four or more standard drinks in a day for women and five or more standard drinks in a day for men) in the past 30 days [22]; smoking an average of at least 5 cigarettes per day; provision of contact information for 2 contacts to assist with follow-up; stable address within 100 km of St. Petersburg; possession of a telephone; interest in cutting down alcohol or tobacco; able and willing to comply with study protocols and procedures.

Participants were excluded from the St PETER HIV study if they were not fluent in Russian or had cognitive impairment resulting in inability to provide informed consent, were pregnant or planning to become pregnant in the next 3 months, or breastfeeding, had unstable psychiatric illness (i.e., answering yes to any of the following: in the past 3 months, active hallucinations, mental health symptoms prompting a visit to the emergency room or hospital, mental health medication changes due to worsening symptoms or the presence of suicidal ideations), had a history of seizures, had acute coronary syndrome within 1 month of enrollment, were taking smoking cessation medications in the past 30 days, had a history of pheochromocytoma, had a history of Buerger’s disease, had a systolic blood pressure greater than 180 mm Hg or diastolic blood pressure greater than 105 mm Hg, were currently taking anti-tuberculosis medications, were currently taking galantamine or physostigmine, had a breath alcohol content (BAC) level of 0.10% or higher or had a known allergy to varenicline or cytisine. For the present study, we excluded patients with values of aspartate transaminase (AST) greater than 300 IU/L.

The study required the collection of blood from each participant at baseline, which was tested for the cluster of differentiation 4 (CD4) cell count, HCV Ab, HIV RNA, AST and alanine transaminase (ALT). For AST and ALT testing, blood was collected in red top tubes and allowed to stand at room temperature for 30 min to allow blood to clot. The tubes were then transferred within 2–4 h after collection to ImmunoBioService (a clinical laboratory in St. Petersburg, Russia) for testing using the Kinetic UV method (Biolabo, Maizy, France).

### 2.3. Independent Variable

The main independent variable was the mean number of cigarettes smoked per day (cpd) in the past 30 days, divided into the following three categories: [5–10 cpd (reference category), 10.1–20 cpd and >20 cpd].

The secondary independent variable was pack-years at study entry. Pack-years isobtained by multiplying the number of packs of cigarettes smoked per day by the number of years smoked (assuming there are 20 cigarettes per pack). We divided the secondary independent variable into three categories [≤20 pack-years (reference category), 20.1–30 pack-years and >30 pack-years]. Both smoking variables were categorized rather thanmodeled as continuous variables as the assumption of a linear relationship with the outcome, advanced liver fibrosis, did not appear to hold. We categorized both smoking variables using thresholds that seemed clinically meaningful.

### 2.4. Outcome Variable

Advanced liver fibrosis was defined as a Fibrosis-4 (FIB-4) index value > 3.25. FIB-4 is a non-invasive index to estimate liver fibrosis that is calculated as follows [23]:

FIB-4 = [age × AST (IU/L)/platelet count (109/L) × ALT (IU/L)^1/2^].

### 2.5. Statistical Analysis

Multivariable logistic regression models were used to evaluate the association between the number of cigarettes per day and advanced liver fibrosis. In addition, we performed a secondary analysis using days of pack-years as the independent variable.

Odds ratios (OR) and 95% confidence intervals were reported from the logistic regression models. The following covariates, selected a priori, were included in the regression: gender; body mass index (BMI); the number of heavy drinking days in the past month; HCV antibody positive; and CD4 count.

We also performed two sensitivity analyses, one that included only patients older than 35 years of age and another that included the presence of self-reported chronic hepatitis B among the covariates.

Two-tailed tests and an alpha level of 0.05 were used for all statistical tests. All analyses were performed using SAS version 9.3 (SAS Institute Inc., Cary, NC, USA).

## 3. Results

A total of 400 participants were included in the present study. Participants were two-thirds male (67.3%), young (median age of 38 years), lean (median BMI of 22), HCV antibody positive (84.5%) and not severely immune suppressed (median CD4 count 351). The prevalence of self-reported chronic hepatitis B, that is, an affirmative answer to the question “Has your doctor ever told you that you have chronic hepatitis B?”, was 24.8%. No patient included in the present study presented acute alcohol-associated hepatitis, and the median AST level was 39 IU/L. The median number of heavy drinking days in the past 30 days was 8 (Interquartile range (IQR): 6–10). The median number of past-month cpd was 20 (IQR: 15–25), 55 participants (13.8%) smoked between 5 and 10 cpd, 221 participants (55.2%) smoked between 10.1 and 20 cpd and 124 participants (31%) smoked more than 20 cpd. The median pack-years was 24 (IQR: 17–31.8); 150 participants (37.4%) smoked less than 20 pack-years, 139 participants (34.8%) smoked between 20.1 and 30 pack-years and 111 (27.8%) participants smoked more than 30 pack-years. The prevalence of advanced liver fibrosis was 11.3% (45/400). Table 1 includes the main characteristics of participants included in this cohort.

In the adjusted logistic regression analysis, we did not observe a significant association between daily cigarette use and advanced liver fibrosis: cigarettes per day [middle (10.1–20.0 cigarettes) vs. lowest (5–10 cigarettes) category (adjusted odds ratio [aOR] (95% confidence interval [CI]): 1.06 (0.40–2.83), highest (>20.0 cigarettes) vs. lowest category aOR (95% CI): 0.65 (0.21–1.99), global *p*-value = 0.62].

The results of the main analysis are shown in Table 2.

In a secondary analysis examining the relationship between smoking pack-years and liver fibrosis, we also did not find evidence of an association: middle (20.1–30 pack-years) vs. lowest category (≤20 pack-years) aOR (95% CI): 0.81 (0.33–1.99), highest category (>30 pack-years) vs. lowest category aOR (95% CI): 0.91 (0.38, 2.19); global *p*-value = 0.58.

The results of the secondary analysis are shown in Table 3.

We performed two different sensitivity regression analyses. The first excluded patients who were younger than 35 years of age, and the second also adjusted for self-reported chronic hepatitis B. The results of those two sensitivity regression analyses were similar to the main regression analysis; that is, we were not able to detect an association between cigarettes smoked or pack-years and the presence of advanced liver fibrosis estimated by FIB-4 in this cohort of Russian PWH who smoked and drank alcohol.

## 4. Discussion

In this Russian cohort of PWH with heavy drinking and daily smoking, we did not detect an association between the number of cigarettes smoked per day and advanced liver fibrosis. In secondary analyses, we did not detect an association between mean pack-years and the presence of advanced liver fibrosis.

Several cross-sectional studies, mostly published in the early 2000s, described an association between tobacco smoking and biopsy-proven liver fibrosis in different cohorts of HCV-infected patients [10]. In the study by Pessione and colleagues, a dose–response association was found between tobacco use and the stage of liver fibrosis, with an odds ratio of 1.2 (95% confidence interval [CI]= 0.6–2.2) for those who smoked ≤15 pack-years and of 1.9 (95% CI= 1.1–3.6) for those who smoked >15 pack-years [24]. In that study, no history of tobacco smoking was the reference category. Dev et al. reported similar results in 2006 in a cohort of 170 participants, where smokers had higher scores of biopsy-proven liver fibrosis, as 21% of smokers had a Metavir score of three versus 14% of non-smokers (*p* < 0.01) [9].

Additionally, other researchers found an association between tobacco use and other measures of liver damage. Namely, in a study by Hezode and colleagues, published in 2003, smoking ≤15 cigarettes per day was associated with the biopsy-proven activity grade, with an OR of 1.2 (95% CI = 0.5–2.8), while smoking >15 cigarettes per day was associated with the necro-inflammation activity grade [A1 = mild versus A2–A3 = marked activity], with an OR of 3.6 (95% CI= 1.5–8.8) [11]. In addition, in a study by Wang that included 886 participants with HCV positive antibodies, tobacco use as a dichotomous variable (Yes versus No) was associated with ALT level elevation with an OR of 1.8 (95% CI = 1.1–2.7), which might also be a surrogate marker of disease activity prior to the occurrence of liver fibrosis [25]. In fact, necro-inflammation is a well-characterized pathological feature of the natural history of chronic HCV infection that usually occurs earlier than liver fibrosis [26]. The more commonly used grading systems in liver biopsies for the evaluation of liver damage in chronic HCV included a measure of necro-inflammation activity and a measure of liver fibrosis [27,28]. It is important to note that those earlier studies involving liver biopsy were performed in the interferon-containing HCV antiviral therapy era, and it is possible that they may suffer from selection bias, given that only very selected patients were evaluated to receive treatment. More recently, a genome-wide association study has also found an association between smoking and liver fibrosis [29]. This study included data from different repositories of European participants, and information about HIV infection and alcohol use was not available [29], which may explain the different results from the present study.

As previously mentioned, studies that have assessed the associations between tobacco use and liver fibrosis in HIV/HCV-coinfected patients are far scarcer. In a study by Wekesa and colleagues performed in Uganda, a history of tobacco use as a dichotomous variable (Yes versus No) was associated with advanced liver fibrosis measured with transient elastography, especially in women treated in rural clinics, with an OR of 2.9 [14]. In that study, the point estimate for the OR among males was lower (1.7), but the association between tobacco use and liver fibrosis was still statistically significant [14].

Consistent with our results, a study of the Canadian Co-infection Cohort did not find an association between tobacco smoking and the progression to advanced liver fibrosis (defined as an APRI score higher ≥ 1.5) or the presence of incident cases of end-stage liver disease [12]. Of note, the prevalence of ever smokers in the cohort was very high (91%), and the study only included cases that did not have advanced liver fibrosis or end-stage liver disease at baseline. The somewhat short follow-up period (1.7 years) with a limited number of outcomes mitigated the ability of the authors to detect an association in a population with a high prevalence of detectable HIV viral load and alcohol use in the past five months [12]. Interestingly, when results were stratified by alcohol use, the point estimates of the odds ratio of the association between ever smoking tobacco and pack-years were higher than one among those who used alcohol and in the direction of an association, while still not significant [12].

Although we were not able to detect an association between cigarette use and liver fibrosis, smoking cessation should be a priority among PWH, given that it is associated with several poor health outcomes. In fact, PWH with comorbidities and/or substance use disorders do not experience the same survival benefit from antiretroviral therapy [30]. Tobacco use is the main modifiable risk factor for the occurrence of cardiovascular disease, and it is a potent carcinogen, responsible for the majority of lung cancers in the developed world, as well as a risk factor for the occurrence and the severity of community-acquired pneumonia [31]. In particular, tobacco use among HIV-infected patients is associated with cardiovascular events [32], cardiovascular deaths and neoplasms [33], as well as with challenges in the management of HIV infection [34], a lack of virological suppression [35], cognitive impairment [36] and aging-related manifestations [37]. In recent years, there has been mounting evidence that tobacco use is a co-factor for the occurrence of liver cancer in end-stage liver disease of different etiologies [13]. It is, therefore, plausible that tobacco might have a deleterious impact on liver disease, but the effect might be difficult to detect with the use of non-invasive indices, which perform better for the detection of advanced liver fibrosis and for the detection of its absence. Whether the impact of tobacco on liver outcomes is due to other pathogenic mechanisms besides liver fibrosis, like oxidative stress or immune dysfunction, remains to be determined. In addition, and as previously mentioned, some of the cross-sectional studies that detected an association between tobacco use and biopsy-proven liver fibrosis did so by accounting for the different stages of liver fibrosis [9,24]. Depending on the grading method used, biopsy-proven liver fibrosis was classified into four or six different stages. Non-invasive indices based on laboratory parameters commonly used in everyday clinical practice, such as FIB-4 or APRI, are not able to classify liver fibrosis in such detail.

This study has important strengths, given that the use of HCV antiviral therapy is still limited in Russia [17], as only 13.7% of the cohort reported having received HCV antiviral therapy, and 9.8% self-reported obtaining a sustained viral response [38]. Therefore, the participants included are a reflection of real-life patients in a cohort before the advent of potent DAA agents [17]. In addition, the use of FIB-4 provides a reliable estimate of advanced liver fibrosis in a resource-limited setting with no access to transient elastography and limited access to liver biopsy ([39]). Given our results, it is unlikely that the patients included in the study have advanced alcohol-associated liver disease. Moreover, the low prevalence of advanced liver fibrosis is probably a reflection of the relatively young age of the participants in the cohort and the relatively conserved number of CD4 cell counts [3]. Therefore, this cohort will likely benefit from modifying risk factors, such as tobacco or alcohol use and from the ongoing use of antiretroviral therapy. In addition, HCV antiviral therapy would be particularly beneficial in a group of patients who still have a mild form of HCV-related liver disease.

However, this study has also several limitations that should be noted. First, all the patients included in the study smoked at least five cigarettes per day, thus the reference category was those who smoked less but who could already have some low-level impact of tobacco use on liver damage. Second, the prevalence of advanced liver fibrosis was somewhat low (only 11.4%), especially in comparison to classic studies of the natural history of chronic HCV infection among PWH [40,41], thus limiting our power to find an association between the independent variable and the outcome if such an association existed. Third, the participants have several competing risks for advanced liver fibrosis, such as heavy alcohol use [7], HIV infection [42] and a high prevalence of HCV coinfection [43] and a low prevalence of HCV antiviral therapy prescription, thus making it harder to detect the additional impact of tobacco use on liver disease. Fourth, we used FIB-4 for the non-invasive estimation of liver fibrosis, which is probably not as accurate as transient elastography. There is also concern that FIB-4 may not be suitable for patients younger than 35 years of age and that, given that it includes the AST level in its formula, FIB-4 values might be impacted by alcohol intake. However, the median AST level in this study population of heavy drinkers was within the normal range. Also, as shown in the results section, the sensitivity analysis that includes only patients older than 35 found similar results to our main analysis. Despite all that, FIB-4 is a reliable method to detect advanced liver disease in patients in whom the performance of a liver biopsy is unlikely [39]. In addition, FIB-4 has been validated against the gold standard of liver biopsy in HIV/HCV-coinfected patients [23]. Moreover, FIB-4 is also a good predictor of mid-term mortality in different cohorts, which is why it has been included in the VACS index [44], an index that strongly predicts mortality in PWH [45]. Fifth, this study was cross-sectional in nature, and the results obtained reflect that we were not able to detect an association between tobacco smoking and liver disease; thus, the findings do not establish a causal relationship. Sixth, our study sample was comprised of HIV-infected heavy-drinking smokers from Russia and has many competing risks for advanced liver disease and also included participants recruited via the snowball method, which may limit the generalizability of our results.

Future studies in this realm should focus on better stratifying patients at risk for alcohol-associated health consequences. An area of interest is the use of more sophisticated methods to estimate liver fibrosis (e.g., transient elastography or shear-wave liver ultrasound) and liver steatosis (e.g., controlled attenuation parameter or magnetic resonance imaging) or the use of direct markers of alcohol use, like Phosphatidylethanol. In addition, in the future, investigators could evaluate different biomarkers of subclinical alcohol-associated damage as well as conduct genomic studies using GWAS to detect polymorphisms associated with poor health outcomes.

In conclusion, in this Russian cohort of PWH with heavy drinking and daily smoking, we did not detect an association between recent cigarette use or mean pack-years and advanced liver fibrosis.

## Figures and Tables

**Table 1:** The characteristics of participants included in this cohort of PWH with heavy drinking and daily smoking.

Factor	N = 400
Female sex [n (%)]	109 (32.7)
Age (years) [median (IQR)]	38 (35–42)
BMI [median (IQR)]	22 (20–24)
AST (IU/L) [median (IQR)]	39 (29–63)
HCV infection [n (%)]	338 (84.5)
CD4 cell count [median (IQR)]	351 (209–542)
FIB-4 > 3.25 [n (%)]	45 (11.3)
Number of cigarettes past 30 days [median (IQR)]	20 (15–25)
Pack-years at baseline [median (IQR)]	24 (17–31.8)
Number of heavy drinking days past 30 days [median (IQR)]	8 (6–10)

IQR = interquartile range; BMI = body mass index; HCV = hepatitis C virus.

**Table 2:** Logistic regression results for the association between past 30-day cigarettes per day (cpd) and advanced liver fibrosis (ALF).

	OR (95% CI)	Global *p*-Value	AOR * (95% CI)	*p*-Value
Cigarettes per day 5–10	1	0.93	1	0.47 ^#^
10.1–20	1.09 (0.42, 2.79)		1.06 (0.40, 2.83)	
>20	0.96 (0.34, 2.66)		0.65 (0.21, 1.99)	
Female sex			0.87 (0.41, 1.85)	0.71
BMI			0.97 (0.87, 1.08)	0.59
HCV infection			6.69 (0.88, 51.12)	0.07
Number of heavy drinking days past 30 days			1.05 (1.00, 1.10)	0.07
CD4 count			0.996 (0.994, 0.998)	<0.01
Age at baseline			1.05 (1.00, 1.11)	0.06

* AOR: Adjusted Odds Ratio; ^#^ global value.

**Table 3:** Logistic regression results for the association between pack-years and advanced liver fibrosis (ALF).

	OR (95% CI)	Global *p*-Value	AOR * (95% CI)	*p*-Value
Pack-years ≤20	1	0.64	1	0.58 ^#^
20.1–30	1.35 (0.64, 2.86)		0.81 (0.33, 1.99)	
>30	1.40 (0.64, 3.07)		0.91 (0.38, 2.19)	
Female sex			0.87 (0.41, 1.87)	0.73
BMI			0.97 (0.87, 1.09)	0.60
HCV infection			7.10 (0.92, 54.61)	0.06
Number of heavy drinking days past 30 days			1.05 (1.00, 1.10)	0.06
CD4 count			0.996 (0.994, 0.998)	<0.01
Age at baseline			1.06 (1.00, 1.13)	0.04

* AOR: Adjusted Odds Ratio; ^#^ global value.

## Data Availability

Data collected for the study are available to interested investigators upon request in the URBAN ARCH Repository: www.urbanarch.org.
